# Association Between E-Cigarette Use and Acculturation Among Adult Immigrants in the United States

**DOI:** 10.1177/1178221819855086

**Published:** 2019-06-13

**Authors:** Yang Wang, Linnea Laestadius, Jim P Stimpson, Fernando A Wilson

**Affiliations:** 1 Public Health Policy & Administration, Joseph J. Zilber School of Public Health, University of Wisconsin-Milwaukee, Milwaukee, WI, USA; 2 Department of Health Management and Policy, Dornsife School of Public Health, Drexel University, Philadelphia, PA, USA; 3 Department of Health Services Research & Administration, College of Public Health, University of Nebraska Medical Center, Omaha, NE, USA

**Keywords:** acculturation, immigrants, electronic cigarettes (e-cigarettes), electronic nicotine delivery systems, smoking, vaping, male, female

## Abstract

Despite a dramatic increase in e-cigarette popularity in recent years, the relationship between acculturation and e-cigarette use among immigrants largely remains unknown. We investigated the association between acculturation, measured by both self-reported English proficiency and length of stay in the United States, and immigrants’ use of e-cigarettes using data from the 2016-2017 National Health Interview Survey. Multivariate logistic regressions were used to examine the associations of acculturation factors with ever and current use of e-cigarettes. We found that high English proficiency increased the odds of ever using e-cigarettes among immigrants (adjusted odds ratios: “well,” 2.22; “very well,” 3.24; with the reference group being “not well”). The association was significant among only men. However, we did not find a significant association between length of stay in the United States and e-cigarette use after adjusting for English proficiency. Future research is warranted to investigate how peer use, family-level factors, country of origin, and marketing strategies jointly influence e-cigarette use among immigrants, especially men.

## Introduction

Since the development of electronic cigarettes (e-cigarettes) in 2004, debate about these products and the larger role of nicotine on health has intensified in the United States.^1^ E-cigarettes allow users to inhale nicotine vaporized by a battery-powered mechanism, rather than burning tobacco leaves. Because they contain neither tar nor carbon monoxide, e-cigarettes have been marketed as a tobacco substitute that is healthier and more socially acceptable in public places through a variety of media channels.^1^ However, e-cigarette aerosol can contain multiple chemicals harmful to the human body,^2^ and its use may serve as a gateway for both combustible cigarette and marijuana use among young adults.^3^ Moreover, e-cigarettes’ long-term health effects and efficacy for smoking cessation remain uncertain.^4,5^

The awareness of e-cigarette products and its consumption has grown substantially. The percentage of US adults who have tried e-cigarettes increased from 1.8% to 15.3% during 2010 to 2016.^6,7^ Current e-cigarette users accounted for 4.5% of US adult population in 2016.^8^ Much of the popularity of e-cigarettes is occurring among younger age groups and non-Hispanic Whites.^9^ Prior research suggests that immigrants are less likely to use tobacco than persons born within the United States.^10–12^ For instance, a recent national study showed that US-born Mexican-Americans had 1.5 times higher odds of being current smokers than recent immigrants with less than 10-year residency in the United States, which indicates that their origin culture might have a protective effect on smoking.^12^ Acculturation describes the process of immigrants maintaining their original culture while developing relationships with the new culture of their host country, resulting in changes in health beliefs and behaviors.^13^ The relationship between smoking tobacco and acculturation, often measured by proxies such as duration in the United States and language proficiency, varies by race/ethnicity and sex.^10,14–16^ For example, Blacks with high proficiency in English were 2.6 times more likely to smoke cigarettes than those with low to moderate proficiency,^10^ whereas language proficiency was not associated with the prevalence of tobacco use among Asian immigrants.^16^ Length of residence in the United States can predict smoking prevalence only among Asian rather than Latino immigrants.^16^ Other research suggests that women are more sensitive to acculturation than men in increasing odds of smoking in Hispanic/Latino communities.^15,17^

Previous research investigated behavioral mechanisms through which immigrants’ initial health advantages erode with acculturation, and highly acculturated immigrants are more likely to engage in unhealthy behaviors.^18^ Wang et al^19^ have examined e-cigarette use by immigration status and the impact of length of stay in the United States on their usage, showing that longer US residency increased the odds of e-cigarette use. However, it failed to include English language proficiency as a key measure and examine the role of sex as a moderator in the acculturation process regarding e-cigarette use. Acculturation processes may vary across sex possibly because of differences in lifestyle, immigration motivation, and so on.^20^ For instance, women who assume more family responsibilities than men may not establish social networks in which e-cigarette users exist, and thus may be less likely to develop the habit of using the product due to peer pressure, even with a high acculturation level. Compared with non-smokers, current and former smokers with adequate English proficiency are more likely to be exposed to e-cigarette advertisement when their craving for cigarettes leads to purchase in retailers or simply seeking information for substitutes online.

To our knowledge, there have been no other prior studies focused on the association between e-cigarette use and acculturation using national data. Thus, it remains unclear if prior findings on immigrants’ use of tobacco are applicable to e-cigarettes, considering that consumers’ sensitivity to its pricing and sale channels may be different from those of combustible cigarettes.^21^ Prior research of immigrant health behaviors suggesting that acculturation is associated with negative health behaviors and health outcomes leads us to hypothesize that acculturation will be positively associated with higher levels of e-cigarette use.^14,18,22^ To test our hypothesis, we used nationally representative data to measure the relationship between acculturation and use of e-cigarettes among US immigrants, as well as its potential heterogeneity across sex and traditional tobacco cigarette smoking status.

## Methods

### Data and sample

We used the most recent 2016-2017 National Health Interview Survey (NHIS) to measure the association between e-cigarette use and acculturation among US immigrants. The NHIS is a nationally representative, in-person annual survey maintained by the National Center for Health Statistics (NCHS) and Centers for Disease Control and Prevention. It provides publicly available, detailed information on demographics, socioeconomic status, health behaviors, access to health care, and other factors. The original dataset consists of 8599 adult immigrants aged 18 years and older. After deleting respondents with missing values (7.5% of the sample), our final analytical sample size was 7954.

### Measures

Two outcome variables in this study included self-reported ever and current use of e-cigarettes. Individuals were defined as e-cigarette ever users based on the question “Have you ever used an e-cigarette, even one time?” Current use of e-cigarettes was based on “Do you now use e-cigarettes?” with possible responses of “every day,” “some days,” and “not at all.” We categorized those reporting “every day” or “some days” as e-cigarette current users.

Respondents reporting that they were born outside of the United States were defined as immigrants. Most were born in Mexico, Central and South America (48.5%), Asia (17.9%), and Europe (11.6%). This study measured acculturation by proxies including both English language proficiency and length of stay in the United States, which have been extensively used in previous studies.^10,14^ NHIS interviewers recorded respondents’ self-reported English proficiency as “very well,” “well,” “not well,” and “not at all.” We categorized those reporting “not well” or “not at all” into 1 group due to small sample sizes. Length of stay in years was categorized into 0 to 4, 5 to 9, and 10 years and above.

Demographic, socioeconomic, health status, and behavior covariates included age in years (18-29, 30-44, 45-64, and 60 and above), sex, race/ethnicity (Hispanic, non-Hispanic White, Black, Asian, and other), educational attainment (less than high school, high school, college, and graduate), marital status (married vs non-married), poverty status (family income below 100%, 100%-199%, and 200% federal poverty line and above), diagnosed asthma (yes vs no), and history of combustible cigarette smoking. Respondents with less than 100 cigarettes smoked in their entire life were defined as non-smokers; those who had smoked 100 or more cigarettes were defined as former smokers if they reported having quit smoking, otherwise current smokers.^23,24^

### Statistical analysis

We characterized the distribution of all measures and performed Pearson χ^2^ tests to determine statistical significance by e-cigarette ever use, including both current use and prior use. We used multivariate logistic regressions to measure the association of e-cigarette ever and current use with language proficiency and length of stay. Considering sex and smoking status as moderators, we further analyzed regression models with interaction terms between length of stay in the United States, English language proficiency, and acculturation proxies. We used Stata 14.0 SE (StataCorp, College Station, TX, USA) to adjust for complex survey design and weights in all the analyses.

## Results

Of the 7954 immigrants, 7.4% (95% confidence interval [CI] = 6.6%-8.1%) reported having used e-cigarettes and the rest 92.6% (95% CI = 91.8%-93.4%) never used e-cigarettes. The distributions of sociodemographic, health condition, and behavior characteristics by e-cigarette use are given in Table 1. Immigrants who ever tried e-cigarettes tended to be younger, male, non-Hispanic White, non-married, current smokers, with college education, family income greater than 200% federal poverty line, and diagnosed asthma. Among men, 10.7% (95% CI = 9.4%-12.1%) and 2.1% (95% CI = 1.5%-2.9%) reported ever and current use of e-cigarettes, respectively, significantly higher than 4.3% (95% CI = 3.6%-5.0%) and 0.7% (95% CI = 0.5%-1.0%) among women (both *P*-values < .001). Regarding acculturation, 68.1% (95% CI = 62.3%-73.3%) of respondents who had ever used e-cigarettes could speak English very well compared with 47.1% (95% CI = 45.0%-49.1%) of those who had never used them (*P* < .001). However, no significant association was found between length of US stay and ever use of e-cigarettes among immigrants (*P* = .204). We found a positive weak correlation between length of stay in the United States and English language proficiency (Cramér’s *V* = 0.078). Almost 52% (95% CI = 49.3%-53.9%) of immigrants who had stayed in the United States for more than 10 years spoke English very well as compared with only 33% (95% CI = 28.9%-38.1%) of those with less than 5 years of residency (results not shown).

**Table 1.** Percentage distributions of sociodemographic, behavior, and health conditions for adult immigrants by e-cigarette use (NHIS 2016-2017).

	Never use (N = 7363)	Ever use (N = 591)	*P*-value
Length of stay in the United States (years)			
0-4	11.0 [9.8, 12.2]	10.4 [7.4, 14.3]	.204
5-9	9.0 [8.1, 10.0]	9.2 [6.0, 13.8]	
10 and above	80.0 [78.4, 81.5]	80.5 [75.5, 84.6]	
English proficiency			
Not well	28.6 [26.7, 30.6]	11.3 [7.7, 16.2]	<.001
Well	24.3 [22.9, 25.8]	20.7 [16.3, 25.8]	
Very well	47.1 [45.0, 49.1]	68.1 [62.3, 73.3]	
Age			
18-29	14.1 [12.9, 15.4]	31.2 [25.7, 37.3]	<.001
30-44	34.6 [33.0, 36.2]	39.5 [34.3, 45.1]	
45-64	36.2 [34.6, 37.9]	25.1 [20.9, 29.9]	
65-	15.1 [14.0, 16.3]	4.2 [2.6, 6.8]	
Sex			
Male	47.0 [45.4, 48.5]	70.4 [65.4, 75.0]	<.001
Female	53.0 [51.5, 54.6]	29.6 [25.0, 34.6]	
Race/ethnicity			
Non-Hispanic White	17.9 [16.4, 19.5]	31.7 [27.0, 36.8]	<.001
Hispanic	47.2 [44.3, 50.1]	40.0 [33.7, 46.7]	
Non-Hispanic Black	9.2 [8.0, 10.5]	6.5 [3.8, 10.8]	
Non-Hispanic Asian	25.1 [23.2, 27.1]	21.1 [16.8, 26.0]	
Non-Hispanic other	0.6 [0.4, 0.9]	0.8 [0.3, 1.8]	
Marital status			
Non-married	35.4 [33.7, 37.1]	54.5 [49.3, 59.6]	<.001
Married	64.6 [62.9, 66.3]	45.5 [40.4, 50.7]	
Educational attainment			
Less than high school	24.8 [23.0, 26.7]	15.2 [11.4, 20.1]	<.001
High school	21.9 [20.4, 23.4]	19.9 [16.3, 24.0]	
College	39.4 [37.7, 41.2]	54.9 [49.1, 60.5]	
Graduate	13.9 [12.6, 15.4]	10.0 [7.5, 13.4]	
Poverty status (%FPL)			
Below 100	17.1 [15.9, 18.5]	12.3 [9.1, 16.4]	.004
100-199	24.6 [23.1, 26.2]	19.7 [15.6, 24.6]	
200 and above	58.2 [56.1, 60.3]	68.0 [62.6, 72.9]	
Cigarette smoking status			
Non-smoker	78.7 [77.4, 79.9]	30.2 [25.4, 35.6]	<.001
Current smoker	6.2 [5.5, 7.0]	42.8 [37.6, 48.1]	
Former smoker	15.1 [14.0, 16.3]	27.0 [22.8, 31.7]	
Asthma			
No	92.2 [91.4, 93.0]	89.1 [85.9, 91.7]	.029
Yes	7.8 [7.0, 8.7]	10.9 [8.3, 14.2]	

Abbreviations: FPL, federal poverty line; NHIS, National Health Interview Survey.

E-cigarette ever use includes both current use and prior use.

Table 2 presents the association between e-cigarette use and acculturation among immigrants using multivariate logistic regressions. Consistent with bivariate analyses, higher English proficiency was associated with increased odds of e-cigarette ever use. Immigrants who spoke English well and very well had, respectively, 2.22 (95% CI = 1.37-3.59) and 3.24 (95% CI = 2.09-5.03) higher adjusted odds of ever using e-cigarettes than those with limited English proficiency. Length of stay in the United States was not associated with ever use of cigarettes, after adjusting for all covariates. We did not find any association between either English proficiency or length of stay in the United States and current use of e-cigarettes. History of tobacco use was a strong predictor of both ever and current use of e-cigarettes.

**Table 2.** Multivariate logistic regression estimates of e-cigarette ever and current use among immigrant adults in the United States (NHIS 2016-2017).

	Ever use	Current use
Length of stay in the United States (years)		
0-4	Ref	Ref
5-9	1.16 [0.60, 2.26]	0.48 [0.08, 2.96]
10 and above	1.56 [0.95, 2.56]	2.49 [0.72, 8.60]
English proficiency		
Not well	Ref	Ref
Well	2.22** [1.37, 3.59]	0.72 [0.24, 2.13]
Very well	3.24*** [2.09, 5.03]	1.38 [0.54, 3.52]
Age		
18-29	Ref	Ref
30-44	0.40*** [0.26, 0.60]	0.48 [0.22, 1.02]
45-64	0.18*** [0.11, 0.27]	0.28** [0.13, 0.62]
65-	0.08*** [0.04, 0.14]	0.04*** [0.01, 0.12]
Sex		
Male	Ref	Ref
Female	0.65** [0.50, 0.85]	0.57 [0.31, 1.05]
Race/ethnicity		
Non-Hispanic White	Ref	Ref
Hispanic	0.77 [0.54, 1.11]	0.57 [0.25, 1.30]
Non-Hispanic Black	0.51* [0.27, 0.98]	0.75 [0.12, 4.72]
Non-Hispanic Asian	0.7 [0.50, 1.00]	1.09 [0.53, 2.25]
Non-Hispanic other	0.52 [0.15, 1.81]	1.03 [0.06, 17.6]
Marital status		
Non-married	Ref	Ref
Married	0.60*** [0.45, 0.80]	0.52* [0.31, 0.90]
Educational attainment		
Less than high school	Ref	Ref
High school	0.98 [0.60, 1.62]	1.05 [0.42, 2.61]
College	1.36 [0.76, 2.42]	1.75 [0.57, 5.41]
Graduate	0.83 [0.44, 1.55]	1.45 [0.39, 5.41]
Poverty status (%FPL)		
Below 100	Ref	Ref
100-199	1.14 [0.69, 1.86]	2.29 [0.84, 6.23]
200 and above	1.55* [1.00, 2.41]	0.86 [0.35, 2.14]
Cigarette smoking status		
Non-smoker	Ref	Ref
Current smoker	23.30*** [16.00, 33.90]	20.90*** [7.79, 56.20]
Former smoker	6.51*** [4.74, 8.93]	11.80*** [4.81, 28.90]
Asthma		
No	Ref	Ref
Yes	1.19 [0.83, 1.70]	1.94 [0.95, 3.96]

Abbreviations: FPL, federal poverty line; NHIS, National Health Interview Survey.

* *P* < .05; **P < .01; ***P < .001.

**Figure 1.**
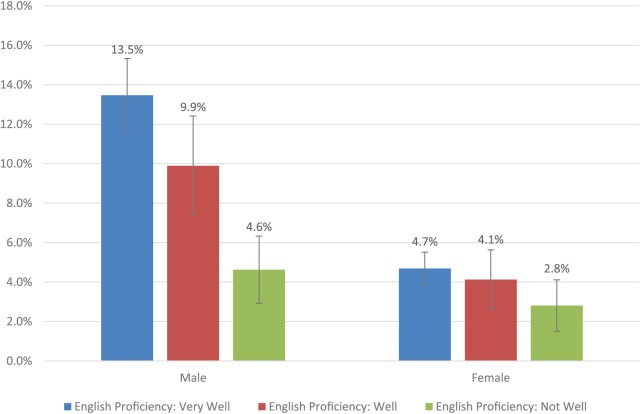
The predicted probability of e-cigarette ever use by sex and English language proficiency (NHIS 2016-2017). NHIS indicates National Health Interview Survey.

The predicted probabilities of e-cigarette ever use by sex and English language proficiency based on regression analyses with interactions between acculturation, sex, and smoking status are provided in Figure 1. Adjusting for all other covariates, 13.5% of male immigrants who spoke English very well used e-cigarettes, which is significantly higher than 4.6% of those who did not speak English very well. However, this association was not observed for female immigrants. English language proficiency was associated with e-cigarette ever use across groups with different smoking status (results not shown). No statistically significant association between length of stay in the United States and e-cigarette ever use was observed by either sex or smoking status (results not shown).

## Discussion

We found that high English proficiency increased the odds of ever using e-cigarettes adjusting for confounding factors. A similar pattern was observed in prior research collecting data from Spanish-speaking patients who were 50% less likely to report having used e-cigarettes than English-speaking counterparts in an emergency department.^25^ Our study suggests that previous findings on tobacco use among immigrants may extend to e-cigarette use.^10,14,17^ However, we did not find a statistically significant association between e-cigarette use and length of stay in the United States. This could be due to the positive correlation between length of stay and language proficiency.^7,10,26,27^ We also found that young respondents were more likely to use e-cigarettes, consistent with prior studies.^9^ We did not find statistical significance for current e-cigarette use possibly because of its fairly low prevalence among US immigrants.

One study reported that peer use increased odds of adolescents’ use of e-cigarettes 1.6 times.^28^ High English proficiency may be associated with more frequent interaction with non-immigrant peers, and thus this may increase the likelihood of acceptance of American mainstream culture and social norms.^10^ This might lead immigrants to try e-cigarettes, which have possibly been stigmatized in their countries of origin.^29^ Furthermore, high English proficiency may make immigrants more susceptible to the large volume of e-cigarette marketing in the United States.^30^ A study from a safety-net hospital in California also corroborated this potential causal pathway, showing that only 50% of non-English-speaking patients were aware of e-cigarettes in contrast to approximately 90% of English-speaking ones.^31^ In our study, there is a stronger, positive association between having ever tried e-cigarettes and English proficiency among men than women. Prior studies indicate that women’s substance use is more likely to increase when exposed to a new culture that is more tolerant of the behavior.^15,17^ Social disapproval of substance use in immigrants’ countries of origin may be stronger for women than for men as compared with the United States.^15^ We speculate that culturally ingrained attitudes toward substance use may protect female immigrants from initiating e-cigarette use through the acculturation process.^18^ This finding reflects the fact that origin cultures may play a dominant role, compared with the adopted culture, in influencing e-cigarette use among female immigrants.

High levels of acculturation were associated with ever use of e-cigarettes across groups with different smoking statuses. However, we do not know if the former smokers in our sample had successfully engaged in cessation through the use of e-cigarettes, or if they simply had a desire to re-initiate nicotine use after previous tobacco cessation. Additional research is needed to explore the extent to which English proficiency is associated with the use of e-cigarettes for successful smoking cessation.

To our knowledge, our study is the first to examine heterogeneous associations between acculturation and e-cigarette use among immigrants by sex, thus highlighting the necessity of targeted interventions to prevent e-cigarette experimentation, such as education programs designed for recent male immigrants; however, it has several limitations. First, e-cigarette use in NHIS is self-reported and has not been validated through other approaches, so our estimates might be subject to recall errors. Respondents may also intentionally provide incorrect answers if they are unwilling to disclose their actual health behavior information due to origin cultural beliefs, which could result in underestimated prevalence of e-cigarette use. Second, we were unable to explore differences in e-cigarette use by country of origin. Publicly available NHIS data only include information on participants’ geographic regions of birth, rather than specific country. Given various cultures within each region, we decided not to conduct further analysis regarding this variable. Third, our data are limited to cross-sectional surveys which could not be used to establish causal relationships, and thus longitudinal data dedicated to immigrant health and behaviors should be collected to address this weakness in the study. Fourth, despite inconsistent definitions and operationalization of acculturation in the existing literature,^32^ efforts have been dedicated to improving acculturation measurements, ranging from simply proxies to multidimensional questionnaires.^33^ For example, a commonly used bi-dimensional instrument captures both adaptation to the host country’s culture and maintaining the original cultural identity.^34^ We were unable to employ detailed measures that capture acculturation levels, such as actual changes in attitudes and behavior, because they are not available in NHIS data. However, the proxies we used are strongly correlated with measurement scales in instruments developed by prior research.^13^ Future studies should address this limitation by longitudinally surveying immigrant populations using widely adopted questionnaires measuring dynamic acculturation processes.

## Conclusions

Our findings suggest that high English language proficiency increases the ever use of e-cigarettes among immigrants, even after adjusting for sociodemographic, smoking behavior, and health status characteristics. Maintenance of cultural norms and limited English proficiency may be preventing immigrants from experimenting with e-cigarettes. This information offers valuable insights for public health professionals working to meet the needs of immigrant communities in the United States. Our study calls for culturally tailored interventions to reduce e-cigarette use among acculturated young immigrants who are not current cigarette smokers. Future research is warranted to investigate how peer use, family-level factors, country of origin, and marketing strategies jointly influence e-cigarette use among immigrants, especially men.
